# Persistent Psychosis due to Single Dose of Ecstasy

**DOI:** 10.7759/cureus.3058

**Published:** 2018-07-27

**Authors:** Sanya Virani, Gulzar N. Daya, Navjot Brainch, Vijaya Padma Kotapati, Deval Zaveri, Saeed Ahmed

**Affiliations:** 1 Psychiatry, Maimonides Medical Center, Brooklyn, USA; 2 Biology, Loyola University, Chicago, USA; 3 Psychiatry, Manhattan Psychiatric Center, New York, USA; 4 Psychiatry, Maimonides Medical Center, New York, USA; 5 Behavioral Health Sciences, Nassau University Medical Center, East Meadow, USA

**Keywords:** mdma, ecstasy, psychosis

## Abstract

Ecstasy, a popular drug among the younger generation, the primary psychoactive component of which is 3,4-Methylenedioxymethamphetamine (MDMA), is rarely known to have acute psychiatric effects and when it does, it is usually short term. We describe a patient who presented to the emergency room in a psychotic state after using ecstasy recreationally. Given his aggressive behavior in the community and risk for self-harm, he was emergently hospitalized to ensure safety. He developed persistent psychotic symptoms (delusions) after one dose of recreational MDMA and the team had the opportunity to observe, monitor, and treat his psychosis. This case along with few other documented cases highlights the gaps in research about the chronic, persistent effects and long-term consequences of MDMA. It also suggests that neuropsychiatric symptoms may not be readily reversible after cessation of use. There is an emphasis on the need for physicians to inquire about MDMA use and include it in toxicology screenings and as a potential differential diagnosis.

## Introduction

Chronic use of 3,4-Methylenedioxymethamphetamine (MDMA), the primary psychoactive component in the drug ecstasy, is popular among young people and has been known to cause several significant medical and psychiatric problems. MDMA is a schedule I drug so very few randomized clinical trials have been conducted to demonstrate its long-term effects and it is difficult to detect in urine so most of the evidence is anecdotal. Some objective studies tie neuropsychiatric symptoms to chronic use.

Short-term psychiatric effects of MDMA seem to be rare and manifest as increased self-confidence, self-acceptance, empathy, love, reduced inhibitions, and heightened sexual sensitivity [1]. Long-term effects of chronic use have been reported to be depression, marked euphoria, anxiety (agitation, panic attacks, simple phobia, loss of appetite, changed sleep patterns and generalized anxiety) and psychosis (paranoid delusions involving jealousy or delusions of grandeur, visual hallucinations, split from reality, depersonalization, hostility, impulsivity) [2, 3].

In this report, we describe a patient who used ecstasy recreationally and presented in a psychotic state.

## Case presentation

Mr. A, a 26-year-old English-speaking immigrant male from Afghanistan, domiciled with his mother and sister at a private residence, had an extensive history of polysubstance use (cannabis, nicotine, alcohol), and was brought into the emergency room (ER) by his family due to agitation, aggression, and a verbal altercation with his neighbors.

Upon initial interview at the ER, he was found to be grossly disorganized, unresponsive to verbal redirection, constantly argumentative, and resisting a full interview and evaluation. He admitted to smoking an unknown amount of marijuana and consuming one pill of ecstasy approximately 12–15 hours earlier. His family reported that they had never seen him in such a state before. His sister stated that prior to the ER visit he displayed uncontrollable aggression to the point of damaging furniture in the home.

She also explained that he had left home early in the morning in a fit of rage, and she had found him on the streets several hours later, banging at the glass windows of a pharmacy with a book. He experienced an episode of psychosis with a particular fixation on a recently read fictitious character, leading to suicidal ideation. Further, the patient was confused regarding his own identity as a human or a fictitious supernatural creature. For the length of time, he went missing and his family also received a phone call from their neighbors stating that he was standing outside their house, making threatening gestures at passersby.

His sister provided more details about his past trauma history, having lived with a father who was alcohol dependent. The patient, along with his family, had been a victim of racial abuse and physical assault, leading to posttraumatic stress disorder (PTSD) and a protectionist savior complex.

In the ER he refused to provide blood and urine samples for toxicology screening, and remained largely uncooperative, displaying more aggression when counseled, punching walls without provocation, and refusing oral medications. The team administered intramuscular Haloperidol 5 mg and Lorazepam 2 mg to ensure his and the staff’s safety. After receiving these medications, he became calmer and provided the blood needed for laboratory tests. All results were within normal limits except for an elevated white blood cell count of 14 (Reference range: 4.5–11.0 x 10^9^/L). The patient was admitted into the inpatient psychiatric unit for further stabilization. Since he was much calmer than when he came into the ER, he did not require seclusion or additional observational orders.

In the psychiatric unit, the patient started becoming aggressive again for the first three days, requiring restraints and intramuscular injections of psychotropic medications on multiple occasions. He was noted to be antagonizing other patients and displaying paranoid behavior towards peers and staff. Interviews for most of his stay were replete with psychotic content and persistent delusions about being a werewolf, while he continued to be irritable and agitated. On subsequent follow-up evaluations, he made it a point to mention almost every day that he had been duped into having himself videotaped while engaging in homosexual activity with a peer.

He was started on a daily oral dose of Paliperidone 3 mg, which was then gradually titrated by 3 mg every week to 9 mg in combination with oral Valproic acid 500 mg twice daily.

He continued to remain psychotic for a week with a minimal response on this regimen. During the second week, he gradually responded and became calmer, less defiant, sociable, and polite in his interactions. By the end of the third week, his psychotic symptoms had subsided with the exception of some delusions.

The team discussed the option of long-acting injections to uphold compliance. The patient agreed and was switched to a long-acting preparation of injectable Invega Sustenna 234 mg, lowered to 156 mg after two weeks.

Toward the end of his hospitalization, the patient revealed that he had consumed ecstasy for the first time. He also said that after taking the pill he started experiencing psychotic symptoms. He admitted to spending $5–10 per day on marijuana, which he inhaled through vaporizers. The patient was discharged with the recommendation to follow up as an outpatient at our clinic and is currently being seen on a monthly basis by an outpatient psychiatrist. His delusions of being a werewolf lasted for a total of five months and eventually subsided. He continues to receive treatment at the clinic and is currently stable.

## Discussion

This report describes how a patient developed persistent psychotic symptoms (delusions) due to one dose of recreational MDMA and was treated successfully with Paliperidone and Valproic acid. The diagnosis of MDMA-induced psychotic disorder was made as symptoms started after the use. An alternate diagnosis of schizophrenia was unlikely because of the complete lack of prodromal symptoms like social withdrawal, anxiety or lack of motivation, and no sustained mood disturbance or impairment in functioning prior to this episode was described. Although the patient admitted to using marijuana as well as ecstasy, the health care providers believed that the clinical presentation was consistent with a persistent psychosis due to MDMA. He had been using marijuana for a long time with no lasting sequelae after than the intended intoxication period.

This case is unusual as most cases to date have only reported psychosis lasting for months and years after chronic MDMA use. The patient, in this case, had been asymptomatic until the one dose of MDMA. Although acute paranoid psychoses are associated with the misuse of amphetamines, persistent psychosis is rare and has been attributed to the precipitation of schizophrenia in most subjects [4]. Earlier case reports have indicated that the MDMA’s capacity for neurotoxicity might induce chronic psychosis de novo [5]. There are very few reports of persistent psychotic symptoms in those who were previously healthy and only used MDMA on sporadic occasions. The cause of MDMA-induced psychotic symptoms is yet to be exactly understood [5]. Persistent damage to serotonergic neurons through rapid release and subsequent depletion of serotonin and dopamine from presynaptic terminals has been thought to be caused by MDMA [6]. It is also known to cause fatal outcomes and two major syndromes most commonly reported as the immediate cause of deaths are hyperthermia (with consequences including disseminated intravascular coagulation, rhabdomyolysis and acute liver and renal failure) and hyponatremia (commonly presenting with confusion and seizures due to cerebral oedema). Other acute harms include cardiovascular and neurological dysfunction leading to seizures and hemorrhage.

In this case, where there is persistent psychosis, it may be related to the issue of long-term neurotoxicity after MDMA exposure. The literature has shown potential for the reversibility of long-term neurotoxicity by comparing the neuropsychiatric statuses of present and former (abstinence periods of approximately two years) ecstasy users [6]. Between current and former users, studies have found little difference in the psychiatric symptom score [7, 8] albeit with a higher degree of anxiety in current users, and lower aggression and persistent depressive symptoms in former users [9]. These findings suggest that neuropsychiatric symptoms may not be readily reversible after cessation of MDMA use [6], which may explain the persistent delusions in this patient. This can be supported by published cases, one of which found that after six months of follow-up, the patient still had mild psychotic symptoms, which were never present before the use of MDMA [10].

It is also noteworthy that these effects resulting from MDMA consumption, in addition to its potential serious medical consequences, are seldom taken into consideration by recreational users. There is now a small but growing body of evidence of persistent psychosis being seen after ingestion of just a single dose of Ecstasy but management strategies with atypical antipsychotics have not yet been established and more research is needed in this direction.

There is also very little literature regarding the treatment of persistent psychosis due to MDMA use. The use of high dose antipsychotics (such as haloperidol and olanzapine) and anticonvulsants (such as diazepam and carbamazepine) has been reported for the treatment of psychosis after MDMA use [3, 4]. The combination of an atypical antipsychotic and mood stabilizer used in the management of this case indicates that further research should incorporate multi-drug combinations of antipsychotics and anticonvulsants.

## Conclusions

This case lends credence to other cases described in the literature, which suggest that MDMA may induce lasting psychotic symptoms. It is also the first of its kind to our knowledge to describe the treatment of MDMA-induced psychosis with Paliperidone. The global use of MDMA and its potential side effects emphasizes the need for physicians to inquire about MDMA use and routinely order toxicology screenings in case of suspicion of use.

MDMA use should be included in the differential diagnosis in patients who present with psychotic symptoms. Once MDMA exposure is suspected, a psychiatrist should be called for collaboration since its use has been associated with serious medical complications. More research should be conducted to determine pathophysiological roles of persistent psychosis after MDMA use and investigate optimal antipsychotic drug combinations for rehabilitation.
